# Stable replication of the EBNA1/OriP-mediated baculovirus vector and its application to anti-HCV gene therapy

**DOI:** 10.1186/1743-422X-6-156

**Published:** 2009-10-02

**Authors:** Hitoshi Suzuki, Norihiko Matsumoto, Tomoyuki Suzuki, Myint OO Chang, Hiroshi Takaku

**Affiliations:** 1 Department of Life and Environmental Sciences, Chiba Institute of Technology, 2-17-1 Tsudanuma, Narashino, Chiba 275-0016, Japan; 2 High Technology Research Center, Chiba Institute of Technology, 2-17-1 Tsudanuma, Narashino, Chiba 275-0016, Japan

## Abstract

**Background:**

Hepatitis C virus (HCV) is one of the main causes of liver-related morbidity and mortality. Although combined interferon-α-ribavirin therapy is effective for about 50% of the patients with HCV, better therapies are needed and preventative vaccines have yet to be developed. Short-hairpin RNAs (shRNAs) inhibit gene expression by RNA interference. The application of transient shRNA expression is limited, however, due to the inability of the shRNA to replicate in mammalian cells and its inefficient transduction. The duration of transgene (shRNA) expression in mammalian cells can be significantly extended using baculovirus-based shRNA-expressing vectors that contain the latent viral protein Epstein-Barr nuclear antigen 1 (EBNA1) and the origin of latent viral DNA replication (OriP) sequences. These recombinant vectors contain compatible promoters and are highly effective for infecting primary hepatocyte and hepatoma cell lines, making them very useful tools for studies of hepatitis B and hepatitis C viruses. Here, we report the use of these baculovirus-based vector-derived shRNAs to inhibit core-protein expression in full-length hepatitis C virus (HCV) replicon cells.

**Results:**

We constructed a long-term transgene shRNA expression vector that contains the EBV *EBNA1 *and *OriP *sequences. We also designed baculovirus vector-mediated shRNAs against the highly conserved core-protein region of HCV. HCV core protein expression was inhibited by the EBNA1/OriP baculovirus vector for at least 14 days, which was considerably longer than the 3 days of inhibition produced by the wild-type baculovirus vector.

**Conclusion:**

These findings indicate that we successfully constructed a long-term transgene (shRNA) expression vector (Ac-EP-shRNA452) using the EBNA1/OriP system, which was propagated in *Escherichia coli *and converted into mammalian cells. The potential anti-HCV activity of the long-term transgene (shRNA) expression vector was evaluated with the view of establishing highly effective therapeutic agents that can be further developed for HCV gene therapy applications.

## Background

Infection by the hepatitis C virus (HCV) is a major public-health problem, with 170 million people chronically infected worldwide [1,2]. The current treatment with combined interferon-ribavirin therapy fails to cure the infection in 30% to 50% of cases [3,4], particularly those with HCV genotypes 1 and 2. Chronic infection with HCV results in liver cirrhosis and can lead to hepatocellular carcinoma [5,6]. Although combined interferon-α-ribavirin therapy is effective for about 50% of the patients infected with HCV, better therapies are needed and preventative vaccines have yet to be developed. In an effort to develop an alternative to combined interferon-ribavirin treatment, we used RNA interference based on short-hairpin RNA (shRNA), which is a powerful tool for suppressing gene function [7]. Small interference RNAs (siRNAs) directed against HCV are likely to successfully block the replication cycle because HCV is an RNA virus and replicates in the cytoplasm of liver cells without integration into the host genome.

The ability of baculoviruses, including *Autographa californica multiple nuclear polyhedrosis virus *(AcMNPV), to infect insect cells has led to their use in multiple protein expression systems [8,9] and as plant insecticides [10]. AcMNPV, the genome of which comprises a circular, double-stranded DNA that contains ~130 Kbp [11] surrounded by a large envelope, infects a variety of mammalian cell types, with the exception of certain hematopoietic cell lines, although its genome does not replicate or integrate into mammalian chromosomes [12,13]. In particular, the inability of baculoviruses to replicate in mammalian cells makes them attractive candidate vectors for *in vitro *gene therapy studies [14,15]. These recombinant vectors contain compatible promoters and are highly effective in infecting primary hepatocyte and hepatoma cell lines, making them very useful tools for studies of hepatitis B and hepatitis C viruses [16-18].

A major limitation of the baculoviral transduction vector, however, is the short duration of transgene expression. Because the baculovirus genome cannot replicate in mammalian cells, it is usually lost or diluted soon after infection. The efficiency of transgene expression must be substantially increased to be applicable for human gene therapy [19]. The Epstein Barr virus (EBV) plasmid is a replicating episomal vector that has been developed to overcome the problem of rapid elimination of intracellularly-delivered plasmid DNA in nonviral vector-mediated gene transfer. EBV is a gamma herpes virus that is maintained as a ~172-kb episome in a small ratio of resting B cells and epithelial cells in most of the human population. EBV induces latent infection in human B cells [20]. When EBV infects cells, the linear and double-stranded genomes are circularized and sustained as a stable episome. The EBV replication system is present at about 1~100 copies per cell [21], and separates by non-covalent attachment to the host chromosome. The EBV replicon vector system has been used to study long-term transgene expression [22,23]. The origin for latent viral DNA replication (OriP) [24] and the latent viral protein Epstein-Barr nuclear antigen 1 (EBNA1) [21] are essential for the replication of EBV [25]. The *EBNA1/OriP *elements have been successfully exploited to achieve durable expression of foreign genes with plasmid- or virus-based expression systems [26-30].

Previously, we demonstrated efficient inhibition of intracellular HCV replication by baculovirus-based shRNA-expressing vectors [31]. This expression system is transient, however, and therefore unable to provide long-term expression of the shRNA. We hypothesized that long-term transgene (shRNA) expression can be significantly improved in mammalian cells using baculovirus-based shRNA-expressing vectors containing *EBNA1/OriP *sequences.

In the present study, we constructed a long-term transgene (shRNA) expression vector (Ac-EP-shRNA452) using the EBNA1/OriP system, which was propagated in *Escherichia coli *and converted into mammalian cells. The potential anti-HCV activity of the long-term transgene (shRNA) expression vector was evaluated with the view of establishing highly effective therapeutic agents that can be further developed for HCV gene therapy applications.

## Results

### Construction of baculovirus transfer vectors carrying shRNA-synthesizing cassettes

**Figure 1:**
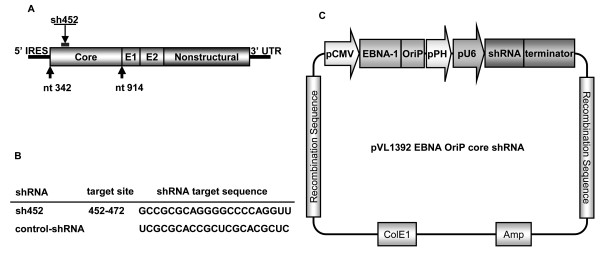
**A Genomic profile of HCV showing both coding and non-coding genes**. **B **HCV core region target sites and sequences used for the design of shRNAs. **C **Construction and schematic representation of EBNA1/OriP baculovirus transfer vector expressing HCV core shRNA.

The core-protein forms the nucleocapsid and modulates gene transcription, cell proliferation, and apoptosis. HCV functions as an mRNA with a single-stranded RNA genome; thus, we hypothesized that cleavage of the core-protein mRNA would inhibit nuclear transport and virus duplication. We previously reported the design of baculovirus vectors expressing shRNA against the following region of the HCV: 452-472, which contains the nuclear localization signal site of the HCV core region (Figure 1A, B) [31]. This vector cannot, however, induce long-term shRNA expression. Therefore, we constructed a long-term transgene shRNA expression vector that contains the EBV *EBNA1 *and *OriP *sequences (Figure 1C). Recombinant baculovirus containing the shRNA genome (Ac-shRNA and Ac-EP-shRNA) was generated by homologous recombination of the transfer vector and linearized baculovirus DNAs (BD Biosciences, San Jose, CA) in Sf9 cells. Viruses were produced at high titers, ranging from 2.0 × 10^8 ^to 4.5 × 10^8 ^pfu/ml.

### Inhibition of HCV RNA replication of EBNA1/OriP baculovirus-mediated shRNA-expression vectors in the HCV replicon

**Figure 2:**
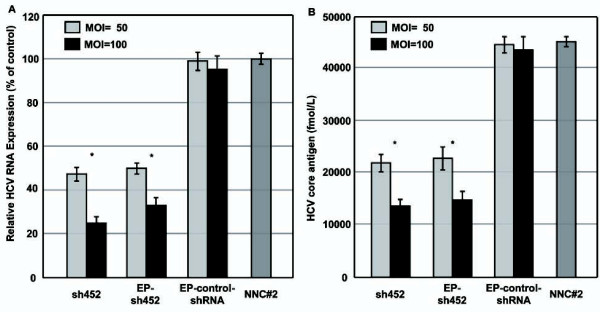
**Inhibition of HCV RNA by EBNA1/OriP and wild-type baculovirus-mediated sh452**. **A **Real time PCR analysis of HCV RNA expression after transduction of HCV full replicon cells (NNC#2, 4 × 10^4 ^cells/well) with an MOI 50 and 100 baculovirus-mediated shRNA. HCV RNA values relative to the scrambled shRNA control are shown. **B **Inhibition of HCV replication by baculovirus-mediated core shRNAs. Ac-shRNAs were used to infect HCV replicons and intracellular HCV core protein levels measured after 3 days by an HCV protein antigen CLEIA assay. Error bars represent standard errors of the mean from three experiments. *p < 0.01.

We investigated whether the intracellular expression of shRNA inhibited viral replication and affected HCV RNA levels in NNC#2 cells. The baculovirus-infection efficiency of NNC#2 cells ranged from 80% to 90% [31]. Real-time reverse transcription polymerase chain reaction (RT-PCR) was used to examine the ability to silence RNA in NNC#2 cells 3 days post-infection. When NNC#2 cells were infected with Ac-shRNAs at a multiplicity of infection (MOI) of 50 and 100, HCV RNA levels were significantly reduced compared with a scrambled shRNA control. Two of the constructs, Ac-shRNA452 (55%, MOI 50; 71%, MOI 100) and Ac-EP-shRNA452 (55%, MOI 50; 67%, MOI 100), inhibited the HCV RNA levels (Figure 2A). In contrast, the control baculovirus vector (Ac-EP-control-shRNA) did not inhibit HCV replication (Figure 2A). These findings indicated that the shRNA had a sequence-specific inhibitory effect on HCV replication. We next used the CLEIA assay to examine whether shRNA against the HCV core protein inhibited viral replication. When NNC#2 cells were infected with Ac-shRNAs at MOIs of 50 and 100, core-protein expression was significantly reduced compared with a non-related shRNA control (Figure 2B). The Ac-EP-control-shRNA baculovirus vectors had no inhibitory effect on HCV replication.

### Enhanced baculovirus-mediated shRNA effects were observed in the presence of EBNA1/OriP

**Figure 3:**
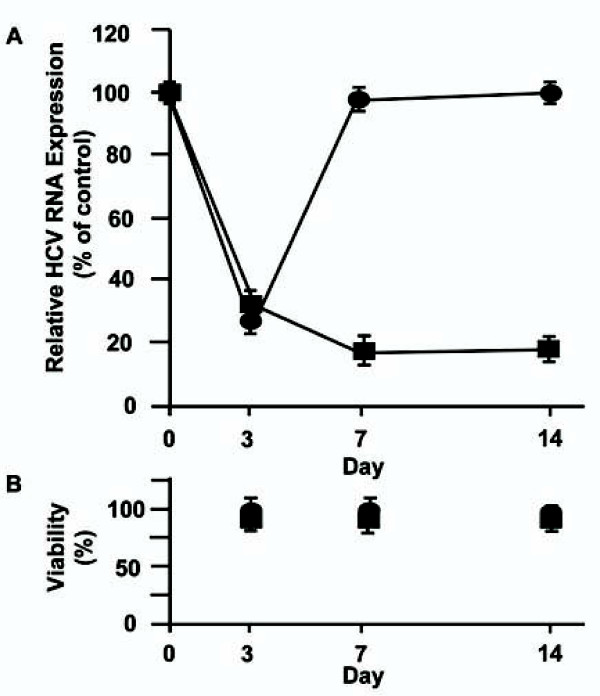
**Long-term inhibition of HCV RNA by EBNA1/OriP and wild-type baculovirus-mediated sh452**. **A **Real-time RT-PCR analysis of HCV RNA expression after transduction of HCV full replicon cells (NNC#2, 4 × 10^4 ^cells/well) with Ac-shRNA452 (MOI = 100 [circle]), Ac-EP-shRNA452 (MOI = 100 [square]). **B **The cytotoxicity of Ac-EP-shRNA452 (square) and Ac-shRNA452 (circle) represented as the percentage reduction of viable Huh-7 cells. A cytotoxicity assay was performed using a BrdU Cell Proliferation ELISA kit according to the manufacturer's instructions (Roche Diagnostics GmbH). The toxicity results are representative of three independent experiments.

To investigate the effect of EBNA/OriP on shRNA expression, we examined the inhibition of HCV replication by Ac-shRNA452 and Ac-EP-shRNA452 in NNC#2 cells for 14 days. When NNC#2 cells were infected with either Ac-shRNA452 or Ac-EP-shRNA452 at an MOI of 100, core-protein expression was significantly reduced compared with a scrambled shRNA control (Ac-EP-control-shRNA) for 3 days (data not shown). Both Ac-shRNA452 and Ac-EP-shRNA inhibited HCV replication for 3 days (Figure 3A). After 3 days, however, cells infected with Ac-shRNA452 exhibited a steady increase in HCV RNA expression while those infected with Ac-EP-shRNA452 continued to have low HCV RNA expression for at least 14 days (Figure 3A). Infection of the NNC#2 cells with recombinant baculovirus vectors containing genetic elements from EBV, EBNA1, and OriP did not induce cellular toxicity, as determined with a bromodeoxyuridine (BrdU)-based colorimetric assay (Figure 3B). These results suggest that HCV RNA expression was more effectively inhibited by the EBNA/OriP baculovirus vector than by the wild-type baculovirus vector.

### Production of EBNA1 protein and siRNA by baculovirus-based shRNA-expressing vectors containing EBNA1/OriP sequences

**Figure 4:**
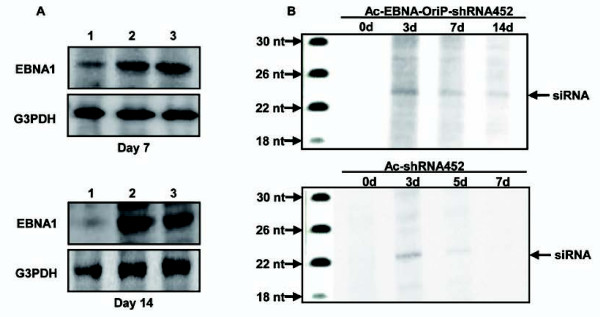
**Detection of EBNA1 protein and siRNA in Ac-EP-shRNA452 infected cells**. **A **Western blot analysis of EBNA1 expression in baculovirus-infected Huh7 cells. Cell lysates were prepared 7 days and 14 days post-infection from cells infected with different viruses. Lane 1: Ac-sh452; lane 2: Ac-EP-sh452; lane 3: Ac-EP-control-shRNA. **B **Expression of siRNA by a baculovirus vector. To demonstrate the intracellular expression of the shRNA construct in the respective siRNA, Huh-7 cells were infected with Ac-EP-shRNA452. The mixture was run on a 15% polyacrylamide TBE urea gel after 3, 7, and 14 days.

We first used Western blot analysis to detect EBNA1 protein in Ac-EP-shRNA-infected cells (Figure 4A). EBNA1 protein was detected in the Ac-EP-shRNA-infected cells. Then, to investigate whether HCV core gene-targeting shRNAs can be digested to mono-specific products of the expected size, siRNAs were analyzed by Northern blot analysis of shRNA-expressing NNC#2 cells. The siRNAs from both Ac-shRNA452 and Ac-EP-shRNA452 yielded products of ~20 nt, which is the expected size of monomeric siRNAs, for 3 days (Figure 4B). The siRNA band in Ac-shRNA452-infected cells, however, became undetectable after 5 days. In contrast, siRNA in Ac-EP-shRNA452-infected cells could be detected for at least 14 days.

## Discussion

There is high demand for the development of effective anti-HCV drugs. Gene silencing by RNA interference is a promising approach to elucidate gene function and to inhibit certain RNA viruses such as HCV [32-34]. Delivery of siRNA to the appropriate cells or tissues, however, is a major challenge. Several approaches have been described for generating loss-of function phenotypes in mammalian systems using siRNA, but these techniques are limited and are not suitable for generating a long-term silencing effect *in vivo *[35,36]. Efficient and safe delivery systems have not yet been established for the suppression of HCV replication. Baculoviruses appear to be useful viral vectors, not only for the abundant expression of foreign genes in insect cells, but also for efficient gene delivery to the hepatoma lines HepG2 and Huh7 [37]. One of the major limitations of the baculoviral transduction vector is the short duration of transgene expression. The EBNA1/OriP system has been widely exploited in many different vectors and cell lines. The findings suggest that the EBNA1/OriP system is effective and useful for long-term and high-level transgene expression.

In this study, recombinant baculovirus vectors containing genetic elements from EBV, EBNA1/OriP, which are essential for the episomal maintenance of the EBV genome in latently infected cells, were constructed and tested for their ability to sustain and express the transgene (enhanced HCV core gene-targeting shRNAs) in HCV replicon cells. The introduction of wild-type or EBNA1/OriP-baculovirus-mediated sh452 into target cells containing HCV replicon RNA induced a dose-related reduction in the level of HCV RNA at 3 days. The effectiveness of the inhibition of HCV replication, however, did not differ under the control of the two different vectors (Ac-shRNA452 or Ac-EP-shRNA452).

To investigate the long-term effect of EBNA/OriP on shRNA expression, we examined the inhibition of HCV replication by Ac-shRNA452 and Ac-EP-shRNA452 in NNC#2 cells for 14 days. Both Ac-shRNA452 and Ac-EP-shRNA inhibited HCV replication for 3 days. After 3 days, however, cells infected with Ac-shRNA452 exhibited a steady increase in HCV RNA expression while those infected with Ac-EP-shRNA452 continued to have low HCV RNA expression for at least 14 days. These recombinant baculovirus vectors containing genetic elements from EBV, EBNA1, and OriP did not induce cellular toxicity in the NNC#2 cells, as determined with a BrdU-based colorimetric assay. The HCV RNA was inhibited by EBNA1/OriP baculovirus-mediated shRNA452 for a longer time by the EBNA1/OriP baculovirus vector than by the wild-type baculovirus vector.

To investigate whether EBNA1/OriP baculovirus-mediated shRNA452 can be digested to mono-specific products of expected size, monomeric siRNAs were performed by Northern blot analysis in AB1-shRNA expressing NNC#2 cells. The shRNAs yielded products ~20 nt, the expected size of monomeric siRNAs, over the long term. Furthermore, EBNA1 protein was also detected in the Ac-EP-shRNA-infected cells. These findings indicated a direct correlation between the level of the virus and siRNA or EBNA1 production.

## Conclusion

The results of the present study indicate that we have successfully constructed a long-term transgene (shRNA) expression vector (Ac-EP-shRNA452) using the EBNA1/OriP system, which was propagated in *Escherichia coli *and converted into mammalian cells. The potential anti-HCV activity of the long-term transgene (shRNA) expression vector was evaluated with the view of establishing highly effective therapeutic agents that can be further developed for HCV gene therapy applications.

## Methods

### Cell culture

NNC#2 (NN/1b/FL) cells [38] carrying a full genome replicon were cultured in Dulbecco's modified Eagle's medium supplemented with 10% fetal bovine serum, non-essential amino-acids, L-glutamine, and 1 mg/ml G418 (Invitrogen, Carlsbad, CA).

### Northern blot analysis

Total RNA was extracted from Ac-shRNA452 infected Huh7 cells using a mirVana™ miRNA Isolation Kit, according to the manufacturer's instructions (Roche Diagnostics GmbH, Mannheim, Germany). Small RNAs (5 μg) were loaded onto a 15% (w/v) polyacrylamide/7 M urea gel. After transfer to a Hybond-N™ nylon membrane (GE Healthcare Bio-Sciences Corp., Piscataway, NJ), synthetic locked nucleic acid (LNA)/DNA oligonucleotides (sh452: 5'-DIG-CCGCGCAGGGGCCCCAGG-3') complementary to the antisense strand of the shRNA452 were used as probes. The membranes were prehybridized for 1 h in DIG EASY hybridization buffer (Roche Diagnostics GmbH) at 60°C and hybridized overnight to the 5'-DIG labeled LNA/DNA probe (10 ng/ml of hybridization buffer). Four post-hybridization washes were performed for 20 min each at 60°C with 2× SSC (1× SSC = 0.15 M NaCl plus 0.015 M sodium citrate-0.1% sodium dodecyl sulfate). LNA/DNA/RNA hybrids were detected using the CSPD chemiluminescent detection system (Roche Diagnostics GmbH).

### Western blot analysis

Cells were lysed in 1× CAT enzyme-linked immunosorbent assay buffer (Roche Diagnostics GmbH). Cell lysates were separated by sodium dodecyl sulfate/polyacrylamide gel electrophoresis and transferred to nitrocellulose membranes, and these were blocked with PVDF Blocking Reagent (TOYOBO, Ohsaka, Japan). The primary antibodies used were monoclonal antibodies against EBNA1 (Acris Antibodies GmbH) and G3PDH (Santa Cruz Biotechnology, Inc., Santa Cruz, CA). Horseradish peroxidase-conjugated anti-goat antibody (Sigma Chemical Co., St. Louis, MO) was used as the secondary antibody.

### RNA purification and real-time RT-PCR

Total RNA was isolated from the cells using a mirVana miRNA Isolation Kit (Ambion, Austin, TX). Real-time RT-PCR was performed using the following primers located in the HCV core region: forward primer (813-833 nt), 5'-CTGGAGGACGGCGTGAATTAT-3'; reverse primer (938-957 nt), 5'-CGTTCGTGACATGGTATATC-3'. HCV-specific RNA was detected by real-time PCR as an increase in SYBR Green I fluorescence on an ABI PRISM 7700 (Applied Biosystems, Foster City, CA). The 18S rRNA housekeeping gene was used as a control for normalization. Each real-time PCR assay was performed in triplicate.

### Cytotoxicity assay

NNC#2 cells (2 × 10^4 ^cells/mL) were seeded into 96-well microtiter plates and incubated in the presence of various concentrations of the test compounds. The dilutions ranged from 1 to 5-fold, and 9 concentrations were examined. All of the experiments were performed in triplicate. After 3 days culture at 37°C in a CO_2 _incubator, cell viability was quantified using a colorimetric BrdU Cell Proliferation enzyme-linked immunosorbent assay according to the manufacturer's instructions (Roche Diagnostics GmbH). The absorbances were read by a microcomputer-controlled photometer (Titertec MultiscanR; Labsystem Oy, Helsinki, Finland) at 405 nm. These values were then translated into percentages per well.

### Baculovirus transfer vector constructs

We designed baculovirus transfer vectors expressing shRNAs against the following region of the HCV core-protein sequence: nucleotides 452-472, which contains the nuclear localization signal site (pU6-core-shRNA452) [31]. The following site in the core region of the common sequence of the HCV strain M1LE (GenBank accession number AB080299) was chosen as the target for the shRNA: 5'-GCCGCGCAGGGGCCCCAGGUU-3' (shRNA452). Sense and antisense strands of shRNA oligonucleotides were synthesized, annealed at 95°C for 3 min, and then slowly cooled in phosphate-buffered saline (pH 7.4, containing 50 mM NaCl). The oligonucleotides contained the loop CCACACC sequence, and KpnI and BamHI ends, which were inserted into a pU6 vector, based on pSV2-neo. A Pol III-type U6 promoter allowed for constant expression of the shRNAs. Fragments of U6-core-sh452, ranging from the *EcoR*I site upstream of the U6 promoter to the BamHI site downstream of the terminating sequence, were sequenced and then inserted into the cloning site of the baculovirus transfer vectors pVL1392 and pVL1393 (BD Biosciences, San Jose, CA) in an opposite orientation to the polyhedrin promoter to create pVL1392-core-shRNA452 and pVL1393-core-shRNA452. A spacer was inserted between the inverted sequences to form a hairpin structure, and to enhance its stability.

The EBV *EBNA1 *and *OriP *gene sequences were obtained from the pCEP4 plasmid (Invitrogen). The *EBNA1/OriP *sequence was digested with restriction enzymes *EcoR*I and *Sal*I, and inserted into the *EcoR*I and *Xho*I sites of pVAX1 (Invitrogen). The cytomegalovirus (CMV) promoter was amplified by PCR using pCEP4 as the template. The CMV promoter was inserted into the *Hind*III and *EcoR*I sites upstream of the *EBNA1/OriP *sequence. The CMV-EBNA1/OriP unit was digested with *Pme*I, and inserted into the *Nae*I site of the baculovirus transfer plasmid pVL1392 (BD Biosciences) to construct pVL1392-EPCMV. Fragments of U6-core-sh452, ranging from the *Not*I site upstream of the U6 promoter to the BamHI site downstream of the terminating sequences, were sequenced and then inserted into the cloning site of the pVL1392-EPCMV baculovirus transfer vector to produce the plasmid pVL1392-EP-shRNA. Scrambled shRNA (control-shRNA) cloned into the same vector was used as a negative control (pVL1392-EP-control-shRNA) in all experiments.

### Preparation of baculoviruses

Recombinant baculovirus containing the shRNA genome (Ac-shRNA) was generated by homologous recombination of the transfer vector and linearized baculovirus DNAs (BD Biosciences) following previously published procedures [39].

### Measurement of HCV core protein

AcU6-HCV-core-shRNAs or Ac-EBNAU6-core-shRNAs were used to infect HCV replicon cells. After 3 days, intracellular HCV core-protein levels were measured using a fully automated HCV core-protein antigen chemiluminescent enzyme immunoassay (CLEIA) according to the manufacturer's instructions [40,41]. The relative chemiluminescence unit was measured and used to determine the concentration of the HCV core antigen according to a standard curve generated using recombinant HCV core antigen. The concentration was expressed in units of femtomole/L (fmol/L). Each CLEIA assay was performed in triplicate.

## Competing interests

The authors declare that they have no competing interests.

## Authors' contributions

HS designed the study, performed all of the experiments, and drafted the manuscript. NM participated in the design of the EBNA1/OriP-baculovirus transfer vector construct experiments. TS and MOOC, participated in the design of recombinant baculovirus experiments. HT conceived the study, participated in its design and co-ordination, and helped to draft the manuscript. All authors have read and approved the final manuscript.
